# Draft Genome Sequence of *Microcystis aeruginosa* CACIAM 03, a Cyanobacterium Isolated from an Amazonian Freshwater Environment

**DOI:** 10.1128/genomeA.01299-16

**Published:** 2016-11-17

**Authors:** Wendel Oliveira Castro, Alex Ranieri Jerônimo Lima, Pablo Henrique Gonçalves Moraes, Andrei Santos Siqueira, Délia Cristina Figueira Aguiar, Anna Rafaella Ferreira Baraúna, Luisa Carício Martins, Hellen Thais Fuzii, Clayton Pereira Silva de Lima, João Lídio Silva Gonçalves Vianez-Júnior, Márcio Roberto Teixeira Nunes, Leonardo Teixeira Dall'Agnol, Evonnildo Costa Gonçalves

**Affiliations:** a Laboratório de Tecnologia Biomolecular, Instituto de Ciências Biológicas (ICB), Universidade Federal do Pará (UFPA), Belém, Pará, Brazil; b Laboratório de Patologia Clínica e Doenças Tropicais, Núcleo de Medicina Tropical, Universidade Federal do Pará, Belém, Pará, Brazil; c Centro de Inovações Tecnológicas (CIT), Instituto Evandro Chagas (IEC), Ananindeua, Pará, Brazil; d Universidade Federal do Maranhão (UFMA), Campus de Bacabal, Maranhão, Brazil

## Abstract

Given its toxigenic potential, *Microcystis aeruginosa* is an important bloom-forming cyanobacterium. Here, we present a draft genome and annotation of the strain CACIAM 03, which was isolated from an Amazonian freshwater environment.

## GENOME ANNOUNCEMENT

*Microcystis aeruginosa* is a bloom-forming freshwater cyanobacterium that may have toxic activity and thus has economic and ecological importance worldwide (1). This species is well known for synthesizing powerful monocyclic heptapeptides known as microcystins, which are a group of hepatotoxins that cause several problems to drinking water supplies, aquatic organisms, human health, and the environment (2).

Genomic data available for cyanobacteria from the Amazonian environment are scarce, with only a few genomes sequenced to date. To improve the genomic data of different cyanobacterial strains, we recovered the draft genome of *Microcystis aeruginosa* CACIAM 03 from the total DNA obtained from a nonaxenic culture. This strain was isolated from a water sample from the Tucuruí hydroelectric power station reservoir (3°50′04.9″S, 49°42′32.2″W) in Pará, Brazil.

After DNA extraction of the cyanobacterial culture, two sequencing runs were performed on the GS FLX 454 (Roche Life Sciences) platform using nonpaired libraries, and one sequencing run was carried out on the Illumina MiSeq platform using a paired-end library with a 150-bp read length. All the raw reads were quality-filtered with a minimum Phred score of 20. A coassembly of all reads was performed by three assembly software programs: Newbler version 2.9 (which was parameterized with minimum overlap of 40 bp, minimum overlap identity of 90%, heterozygote mode, and extended low-depth overlap options on), CLC Genomics Workbench (http://www.clcbio.com) (default parameters), and SPAdes version 3.9 (3) (with parameter flag for metagenome and *k*-mer sizes of 21, 33, 55, 77, 99, and 127). These assemblers produced 1,484, 14,250, and 5,742 scaffolds, respectively.

MaxBin version 2.2.1 (4) was used to bin each set of assembled scaffolds. To classify taxonomically the obtained bins, we performed a BLASTp analysis for each bin in the sequences containing hidden Markov models for essential genes identified by MaxBin version 2.2.1 against the NCBI nonredundant database. The results were visualized on MEGAN 5 (5). The bins identified as *M. aeruginosa* were integrated and subsequently processed using the hybrid assembly program CISA version 1.3.1 (6), producing 249 scaffolds (>1.852 bp length) with an *N*_50_ value of 33,157, a total of 5.0 Mb, and a GC content of 42.9%.

The scaffolds were annotated by the NCBI Prokaryotic Genome Annotation Pipeline (7). This process identified 4,197 coding sequences, 44 tRNA genes (including 1 tmRNA), 8 rRNA genes, 614 pseudogenes, and 4 noncoding RNA genes.

Preliminary analysis with antiSMASH version 3.0 (8) revealed gene clusters involved in the nonribosomal biosynthesis of microcystin, aeruginosin, and terpene, as well as gene clusters involved in ribosome synthesis of peptides such as yersiniabactin (bacteriocin), micropeptin (microviridin), microcyclamide (cyanobactin), and microcyclamide (bacteriocin), which are of great biotechnological value.

This report can improve the genomic data about *M. aeruginosa* by including the first genome of this species obtained from an Amazonian environment.

### Accession number(s).

This whole-genome shotgun project has been deposited at DDBJ/EMBL/GenBank under the accession number MCIH00000000. The version described in this paper is the first version, MCIH01000000.
